# LINC00961 inhibits the migration and invasion of colon cancer cells by sponging miR‐223‐3p and targeting SOX11

**DOI:** 10.1002/cam4.2850

**Published:** 2020-02-11

**Authors:** Haixia Wu, Yuedi Dai, Dexiang Zhang, Xiaoyu Zhang, Zhiyun He, Xiaojun Xie, Chudong Cai

**Affiliations:** ^1^ Department of Medical Oncology Cancer Hospital of Fudan University Minhang Branch, Shanghai China; ^2^ General Surgery Department Zhongshan‐Xuhui Hospital Affiliated to Fudan University Shanghai China; ^3^ Department of General Surgery Division of Gastrointestinal Surgery Huai'an Second People's Hospital The Affiliated Huai'an Hospital of Xuzhou Medical University Huai'an China; ^4^ Colorectal Surgical Department Lanzhou University Second Hospital Lanzhou China; ^5^ Department of General Surgery The First Affiliated Hospital of Shantou University Medical College Shantou China; ^6^ Department of General Surgery Shantou Central Hospital The Affiliated Shantou Hospital of Sun Yat‐Sen University Shantou China

**Keywords:** colon cancer, invasion, LINC00961, migration, miR‐223‐3p, SOX11

## Abstract

Long noncoding RNAs play essential roles in colon cancer tumorigenesis. This study aimed to explore the potential function and molecular mechanisms of LINC00961 in colon cancer. qPCR results showed that LINC00961 was downregulated in colon cancer cells and tissues. Functional assays demonstrated that LINC00961 suppressed the migration and invasion of colon cancer cells in vitro. LINC00961 functioned as an endogenous sponge for miR‐223‐3p in colon cancer cells. SOX11 was confirmed as a target gene of miR‐223‐3p. The effect of miR‐223‐3p on colon cancer cells was then investigated. MiR‐223‐3p inhibition enhanced their migration and invasion. The effect of SOX11 on colon cancer cells was studied. SOX11 overexpression inhibited the invasion of colon cancer cells. LINC00961 acted as an anti‐oncogene and upregulated SOX11 expression by functioning as a miR‐223‐3p sponge. This research revealed the molecular mechanism of LINC00961 in colon cancer. LINC00961 might act as a potential diagnostic biomarker and therapeutic target for further clinical treatments.

## Graphical abstract

LINC00961 was downregulated in colon cancer and functional assays demonstrated that LINC00961 suppressed the migration and invasion of colon cancer cells in vitro. LINC00961 acted as an anti‐oncogene and upregulated SOX11 expression by functioning as a miR‐223‐3p sponge.

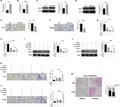

## 1 INTRODUCTION

Colon cancer is the third‐most frequent cancer worldwide.^1^, ^2^ Although great treatment advancements have been achieved, the high frequency of recurrence in patients with colon cancer remains the major problem in clinical practice.^3^, ^4^ Effective treatments for the metastasis of colon cancer cells are limited. No satisfactory therapy is available for patients with distant metastasis. Numerous studies have been performed to investigate the mechanisms of colon cancer recurrence, which is the cornerstone of the solution of clinical questions, and solve this clinical problem.^5^

Long noncoding RNAs (lncRNAs) are a vital group of noncoding RNA molecules that are longer than 200 nucleotides.^6^, ^7^ The rapid development of RNA genomics has highlighted the oncogenic or anti‐oncogenic role of lncRNAs in colon cancer.^8^ SLCO4A1‐AS1 accelerates the colorectal cancer development through Wnt signaling pathway.^9^lncRNA PVT1 facilitates the tumor progression in gallbladder cancer via the miR‐143/HK2 axis.^10^ lncRNA ZNFX1‐AS1 enhances the invasion colorectal cancer cells of miR‐144/EZH2.^11^ Long intergenic nonprotein coding RNA 961 (LINC00961, gene ID: 158 376) is a novel lncRNA, which is a vital regulator in multiple tumors. LINC00961 suppresses the tumor cells invasion by β‐catenin signaling pathway in tongue tumor.^12^ However, the biological function and potential mechanism of LINC00961 in colon cancer are completely unknown.

MicroRNAs (miRNAs), which are small noncoding RNAs of 20‐25 nucleotides in length, regulate the expression of downstream targets via post‐transcriptional modulation.^13^ MiR‐198 facilitates the colorectal cancer cells apoptosis and growth through regulating the ADAM28/JAK‐STAT pathway.^14^ Moreover, miRNA‐124‐3p restrains the bladder cancer progression via downregulation of ITGA3. Emerging evidence has suggested that the lncRNA‐miRNA‐mRNA‐regulating network is involved in tumor progression. LncRNA FBXL19‐AS1 strengthens the breast cancer cells invasion and growth abilities by sponging miR‐718.^15^ LncRNA ZEB1‐AS1 promotes TGF‐β1‐induced invasion of bladder tumor cells via targeting the miR‐200b/FSCN1 pathway.^16^ LINC00152 suppresses the development of esophageal carcinoma via sponging miR‐153‐3p and targeting FYN.^17^

In this research, LINC00961 was downregulated in colon cancer. LINC00961 suppressed cell migration and invasion in vitro. The underlying mechanism of LINC00961 in colon cancer was explored. Our study confirmed that LINC00961 suppressed the migration and invasion of colon cancer cells through the miR‐223‐3p/SOX11 axis. 

## 2 MATERIAL AND METHODS

### 2.1 Tissue specimens

Tumor and normal tissues were obtained from patients who were diagnosed with colon cancer and who had undergone surgery at Second People's Hospital of Huai'an. Twenty colon cancer tissues were collected 2016 to 2017 and were frozen in liquid nitrogen. This research was approved by the Second People's Hospital of Huai'an Research Ethics Committee.

### 2.2 Cell culture

Colon cancer lines were obtained from Shanghai Institute of Cell Biology (Shanghai, China). Four colon cancer lines, namely, HT29, SW480, SW620, and DLD1, and the normal colon cell line FHC were cultured in an incubator (37°C, 5% CO_2_) and in RPMI1640 (Gibco, USA) supplemented with 10% FBS (Gibco, USA).

### 2.3 Cell transfection

PcDNA3.1 vector was chosen as the supporter of overexpression SOX11 in which full length sequence was cloned into it. The empty pcDNA3.1 vector was used as the negative control. The lentiviral vector for LINC00961 was constructed by Jikai Gene (Shanghai, China). In order to overexpress or inhibit miR‐223‐3p, a mimic or inhibitor of miR‐223‐3p was purchased from RiboBio (Guangzhou, China).

### 2.4 Isolation of nuclear and cytoplasmic RNA

Isolation of nuclear and cytoplasmic RNA was actualized according to the literature.^18^ The cytoplasm and nuclear RNAs of cells were separated and extracted using a nuclear and cytoplasmic RNA purification kit (Norgen, USA). qPCR assay was performed to detect the isolated RNA.

### 2.5 Transwell migration and invasion assay

Transwell migration and invasion assay was performed in accordance with previously described methods.^19^ For the Transwell migration assays, the transfected HT29 and SW480 (N = 6 × 10^3^) cells were plated in top chambers with a noncoated membrane. For the invasion assays, the transfected HT29 and SW480 (N = 10 × 10^3^) cells were plated in top chambers with a coated membrane. The number of invading colon cancer cells was counted after they were fixed with 4% paraformaldehyde.

### 2.6 Quantitative reverse transcription polymerase chain reaction

RNA was isolated using TRIzol reagent (Life Technologies, US). SYBR Green qRT‐PCR was conducted to measure mRNA expression levels by using an ABI7300 real‐time PCR machine. miR‐223‐3p, SOX11, and GAPDH expression levels were examined using the following specific primers: 5′‐ CGCUAUCUUUCUAUUAACUGACCAUAA‐3′ and 5′‐ CGCUAUCUUUCUAUUAUGACUCCAUAA‐3′, 5ʹ‐AGCAAGAAATGCGGCAAGC‐3ʹ and 5ʹ‐ATCCAGAAACACGCACTTGAC‐3ʹ, 5ʹ‐GGAGCGAGATCCCTCCAAAAT‐3ʹ and 5ʹ‐GGCTGTTGTCATACTTCTCATGG‐3ʹ.

### 2.7 Western blot

The protein (15‐25 ug) extracted from the sample was utilized for Western blot analysis. Proteins from lysed cells were separated by 10% SDS‐PAGE and transferred to nitrocellulose membranes, then blocked for 2 h. Next, the membranes were incubated overnight with primary antibodies, followed by horseradish peroxidase (HRP)‐conjugated secondary antibodies. The antibody utilized in this research included anti‐SOX11 (1:1000; HK), and GAPDH (1:1000; USA) was utilized as the loading control.

### 2.8 Dual‐luciferase reporter assay

Luciferase reporter assay was actualized according to the literature.^20^, ^21^ The wt‐pmirGLO‐LINC00961 and wt‐pmirGLO‐SOX11 reporters and corresponding mutated vectors were established by Jing Kairui (Wuhan, China). In addition, wt/mut‐pmirGLO‐LINC00961 or wt/mut‐pmirGLO‐SOX11 reporter was co‐transfected into colon cancer cells with mimic‐miR‐223‐3p or inhibitor‐miR‐223‐3p (100 nmol L^‐1^) with Lipofectamine 2000. According to standard protocols, luciferase activity of cells with transfection was measured by fluorescence detector (Toyo Ink, Tokyo, Japan).

### 2.9 In vivo study

The HT29 cell line stably overexpressing LINC00961 was established. A lung metastasis mice model was also established with the intra‐splenic injection of 4 × 106 stably overexpressing LINC00961 or LV‐NC cells. After 21 days, lung colonization capacity was evaluated. The number of lung metastatic foci was counted via H&E staining. The study was approved by the ethic committee of The Affiliated Huai'an Hospital of Xuzhou Medical University and The Second People's Hospital of Huai'an. The experiments were performed in accordance with the NIH requirement.

### 2.10 Statistical analysis

Data were statistically analyzed using Student's t‐test and one‐way ANOVA in GraphPad Prism 5.0 and SPSS 13.0. Statistical data were expressed as mean ± standard deviation (s.d.). Differences were considered significant at *P* < .05.

## 3 RESULTS

### 3.1 The downregulated LINC00961 inhibited the migration and invasion of colon cancer cells

**Figure 1:**
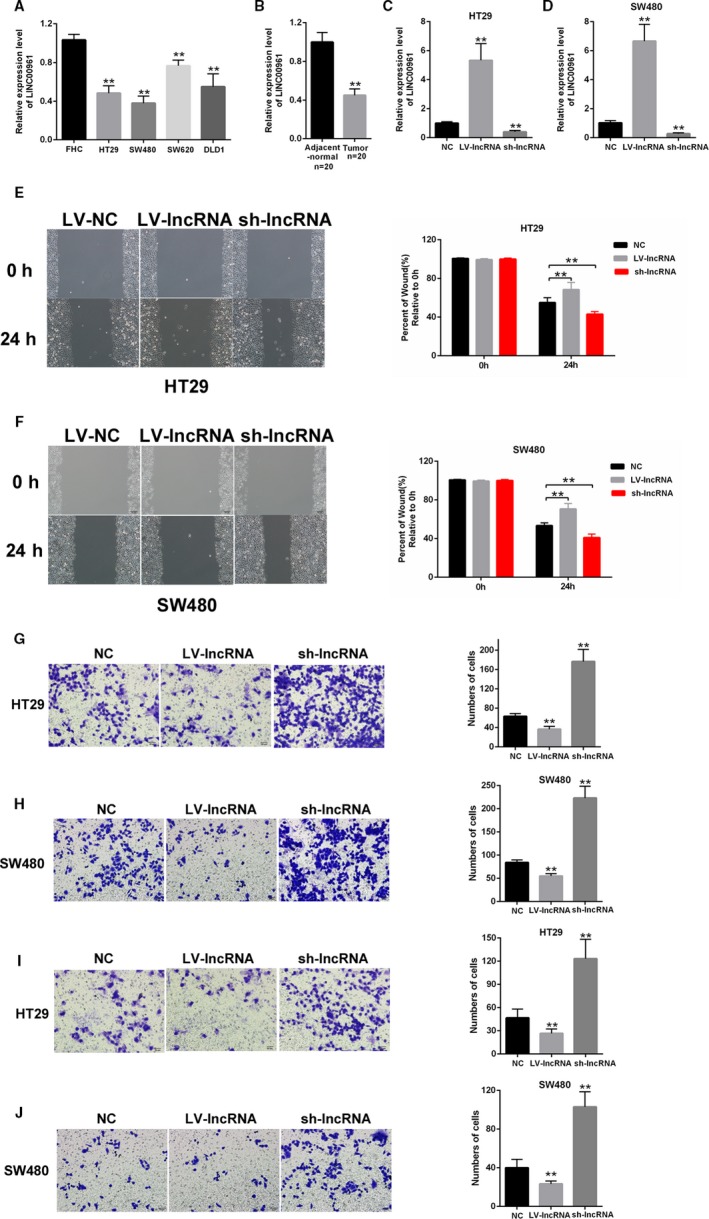
Downregulated LINC00961 inhibited the migration and invasion of colon cancer cells. (A) LINC00961 expression levels were downregulated in colon cancer cell lines compared with those in normal lines. (B) LINC00961 expression levels decreased in colon cancer compared with those in tumor‐adjacent normal pairs (N = 20). (C and D) The efficiencies of LV‐LINC00961 and sh‐LINC00961 were determined through qRT‐PCR. (E and F) Wound‐healing assays indicated that LINC00961 overexpression suppressed cells migration and LINC00961 knockout enhanced cells migration. (G) Cell migration in HT29 was inhibited by LINC00961 overexpression but was enhanced by LINC00961 knockout. (H) Cell migration in SW480 was inhibited by LINC00961 overexpression but was enhanced by LINC00961 knockout. (I) Cell invasion in H29 was decreased by LINC00961 upregulation but was increased by LINC00961 downregulation. (J) Cell invasion in SW480 was decreased by LINC00961 upregulation but was increased by LINC00961 downregulation. Each experiment was performed in triplicate. **P < .01 vs control

LINC00961 expression was decreased in colon cancer cell lines, namely, HT29, SW480, SW620, and DLD1, compared with normal colon cell FHC (Figure 1A). The expression level of LINC00961 was downregulated in colon cancer tissues than in paired adjacent tissues (N = 20) (Figure 1B). Overexpression and knockdown assays were performed in HT29 and SW480 cells to explore the biological functions of LINC00961. The efficiency of LV‐LINC00961 and sh‐LINC00961 was determined through qRT‐PCR (Figure 1C,1). Then H29 and SW480 were transfected with LV‐LINC00961 or sh‐LINC00961 and wound‐healing assays were performed. Wound‐healing assay results indicated that LINC00961 overexpression weakened the cell migration and silencing of LINC00961 promoted the cells migration in HT29 and SW480 (Figure 1E,1). Moreover, transwell assays were performed in colon cancer cells. The cell migration in HT29 and SW480 was inhibited by LINC00961 upregulation but was enhanced by LINC00961 downregulation (Figure 1G–H). The cell invasion in HT29 and SW480 was suppressed by LINC00961 overexpression but was promoted by LINC00961 knockout (Figure 1I–J). These results demonstrated that LINC00961 inhibited the colon cancer cells migration and invasion.

### 3.2 MiR‐223‐3p was predicted as a direct target of LINC00961

**Figure 2:**
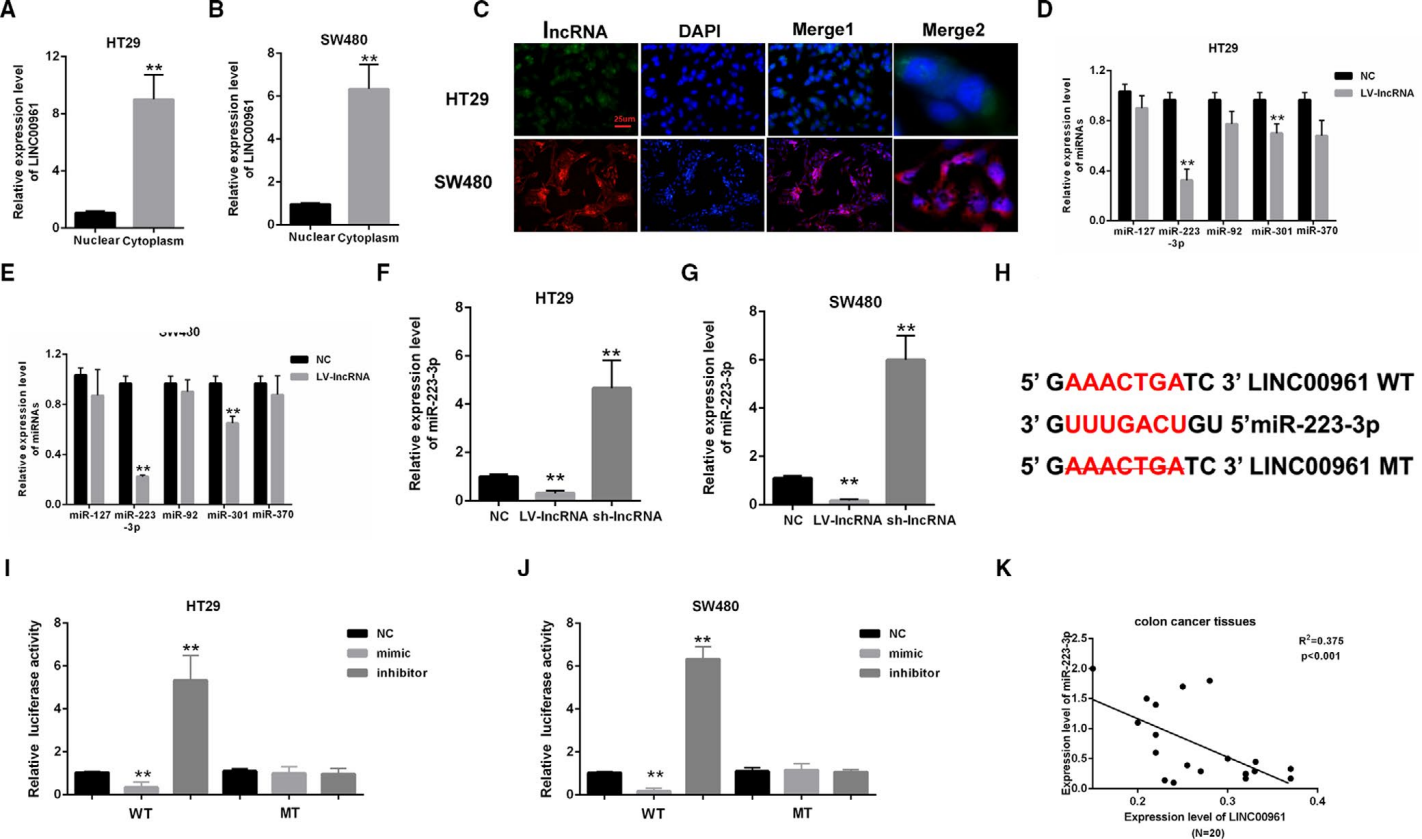
MiR‐223‐3p was predicted as a direct target of LINC00961. (A‐C) qPCR and FISH results indicated that LINC00961 was mainly expressed in the cytoplasm of HT29 and SW480 cells. (D and E) Five candidate miRNAs were screened by qPCR. miR‐223‐3p expression level is the lowest compared with negative control among candidate miRNAs. (F and G) qPCR results showed that miR‐223‐3p expression in HT29 and SW480 was decreased by LINC00961 overexpression but was increased by LINC00961 knockout. (H) The direct binding sites between LINC00961 and miR‐223‐3p were presented. (I and J) Luciferase reporter assay was performed to confirm the direct binding relationship between LINC00961 and miR‐223‐3p. (K) There is negative correlation between LINC00961 and miR‐223‐3p in colon cancer tissues. All experiments were performed three times. **P < .01 vs control

LINC00961 was mainly localized in the cytoplasm as measured by qPCR and fluorescence in situ hybridization (FISH) in HT29 and SW480 (Figure 2A–C). Five candidate miRNAs predicted by GEO DataSets (https://www.ncbi.nlm.nih.gov/pubmed/), including miR‐127, miR‐223‐3p, miR‐92, miR‐301, and miR‐370, were screened by qPCR in HT29 and SW480 transfected with LV‐ LINC00961 or LV‐NC. QRT‐PCR results indicated that miR‐223‐3p expression level is the lowest compared with negative control among candidate miRNAs (Figure 2D,2). MiR‐223‐3p was chosen as a candidate for LINC00961. QPCR assay indicated that the miR‐223‐3p expression level in HT29 and SW480 was decreased by LINC00961 overexpression but was increased by LINC00961 silencing (Figure 2F,G). The direct binding sites between LINC00961 and miR‐223‐3p are presented in Figure 2H. Dual‐luciferase reporter assays were performed in HT29 and SW480. The results indicated that the luciferase activity of LINC00961 was inhibited by mimic‐miR‐223‐3p but was upregulated by inhibitor‐miR‐223‐3p. However, the luciferase activity of mutant‐type reporter gene was not inhibited or increased miR‐223‐3p (Figure 2I,J). Finally, the expression relationship between LINC00961 and miR‐223‐3p was determined in colon cancer tissues. qPCR results showed that there is negative correlation between LINC00961 and miR‐223‐3p (Figure 2K). These results suggested that miR‐223‐3p was a direct target of LINC00961.

### 3.3 MiR‐223‐3p promoted the migration and invasion of colon cancer cells

**Figure 3:**
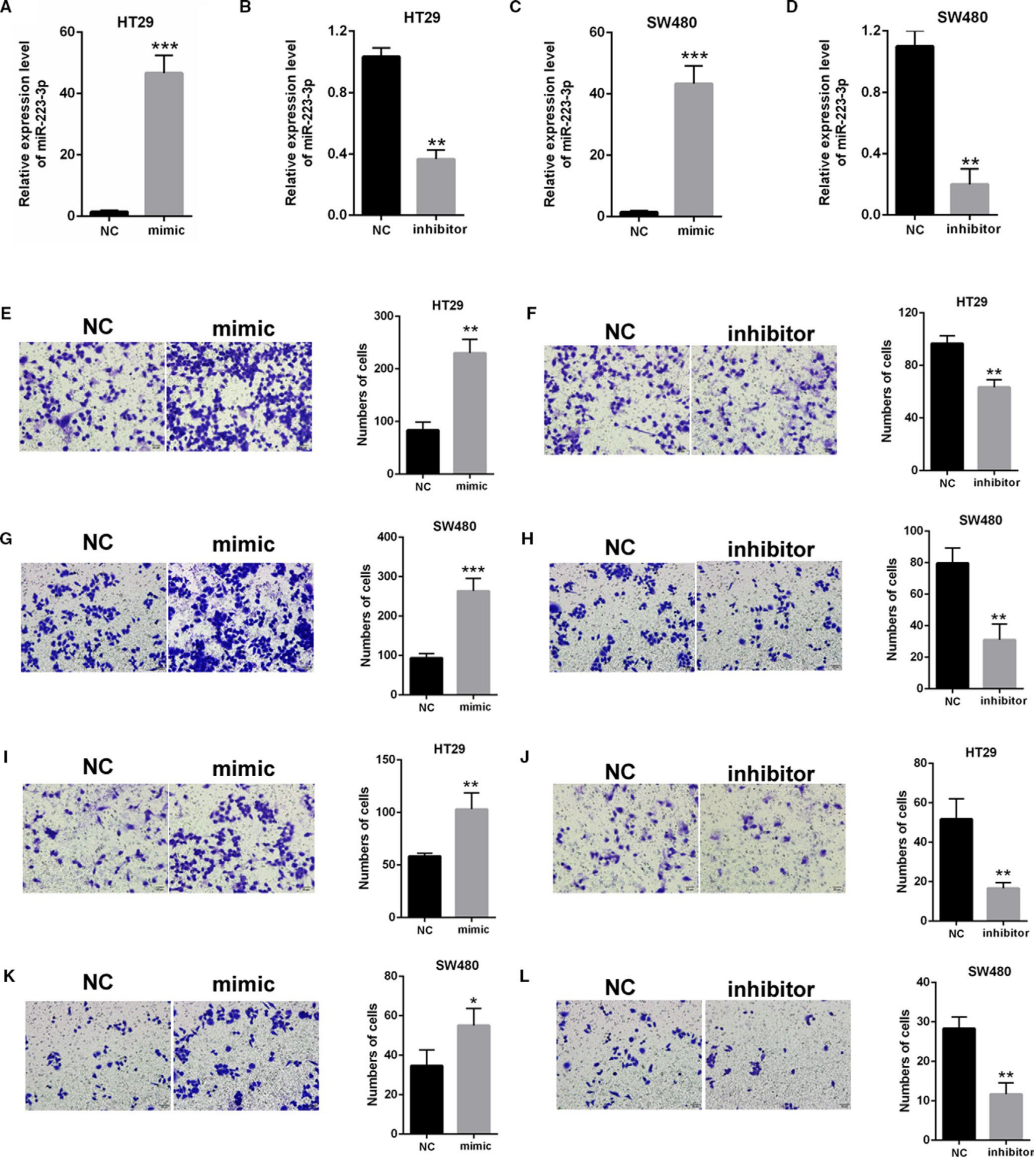
Effects of miR‐223‐3p on the migration and invasion of colon cancer cells. (A‐D) The efficiency of mimic‐ and inhibitor‐miR‐223‐3p was determined by qPCR in HT29 and SW480. (E‐H) The effect of mimic‐ and inhibitor‐223‐3p on HT29 and SW480 cell migration by Transwell assays. Cell migration was enhanced by mimic‐223‐3p but was inhibited by inhibitor‐223‐3p. (I‐L) The effect of mimic‐ and inhibitor‐223‐3p on HT29 and SW480 cell invasion by Transwell assays. Cell invasion was increased by mimic‐223‐3p but was decreased by inhibitor‐223‐3p. Each experiment was performed in triplicate. **P < .01,***P < .001 vs control

The effect of miR‐223‐3p on colon cancer was investigated. The efficiency of mimic‐ miR‐223‐3p and inhibitor‐miR‐223‐3p in HT29 and SW480 was determined by qPCR (Figure 3A–D). The cell migration in HT29 and SW480 was enhanced by mimic‐miR‐223‐3p but was suppressed by inhibitor‐miR‐223‐3p (Figure 3E–H). Mimic‐ and inhibitor‐miR‐223‐3p facilitated and decreased the cell invasion of both HT29 and SW480 respectively (Figure 3I–L). Therefore, miR‐223‐3p enhanced the colon cancer cell migration and invasion.

### 3.4 SOX11 was confirmed as a direct target gene of miR‐223‐3p

**Figure 4:**
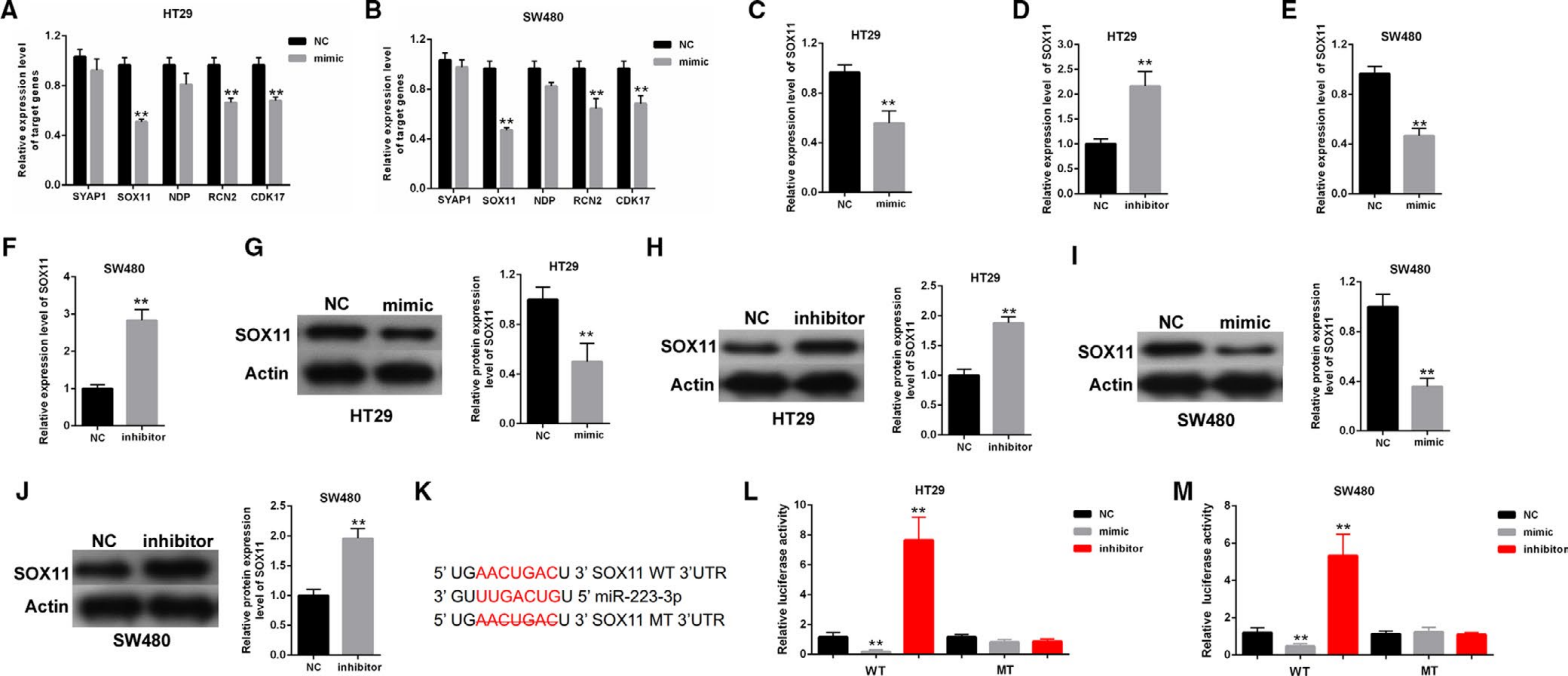
SOX11 was a direct target gene of miR‐223‐3p. (A and B) Five candidate genes were screened by qPCR. SOX11 expression level is the lowest compared with negative control among candidate genes. (C‐F) qPCR results indicated that the mRNA expression level of SOX11 in HT29 and SW480 was decreased by mimic‐223‐3p but was increased by inhibitor‐223‐3p. (G‐J) Western blot results showed that the protein expression level of SOX11 in HT29 and SW480 was inhibited by mimic‐223‐3p and was enhanced by inhibitor‐223‐3p. (K) The direct binding sites between miR‐223‐3p and SOX11 are presented. (L and M) Luciferase reporter assay was performed to confirm the direct binding relationship between miR‐223‐3p and SOX11. All data analyses were repeated thrice independently. **P < .01 vs control

Five candidate genes predicted by Targetscan (http://www.targetscan.org/vert_72/), including SYAP1, SOX11, NDP, RCN2, and CDK17, were screened by qPCR in HT29 and SW480 transfected with mimic or mimic‐NC. QRT‐PCR results indicated that SOX11 expression level is the lowest compared with negative control among candidate miRNAs (Figure 4A,4). In order to explore the function of miR‐223‐3p in colon cancer, qPCR and western blot were conducted to confirm the regulation of SOX11 in both cells with miR‐223‐3p mimic or inhibitor treatment. qPCR results showed that the mRNA expression levels of SOX11 in HT29 and SW480 were decreased by mimic‐miR‐223‐3p but was increased by inhibitor‐miR‐223‐3p (Figure 4C–F). Western blot results indicated that the protein expression levels of SOX11 in colon cell lines were inhibited by miR‐223‐3p overexpression but was promoted by miR‐223‐3p silencing (Figure 4G–J). As shown in Figure 4K, the 3’‐UTR of SOX11 contains a putative binding site of miR‐223‐3p. Luciferase reporter assay results indicated that the luciferase activity of wild‐type SOX11 was suppressed by mimic‐miR‐223‐3p but was enhanced by inhibitor‐miR‐223‐3p. However, the luciferase activity of mutant‐type reporter gene remained unchanged by mimic‐223‐3p or inhibitor‐223‐3p (Figure 4L,M). The results indicated that SOX11 was a direct target gene of miR‐223‐3p.

### 3.5 LINC00961 regulated SOX11 expression and inhibited colon cancer invasion by sponging miR‐223

**Figure 5:**
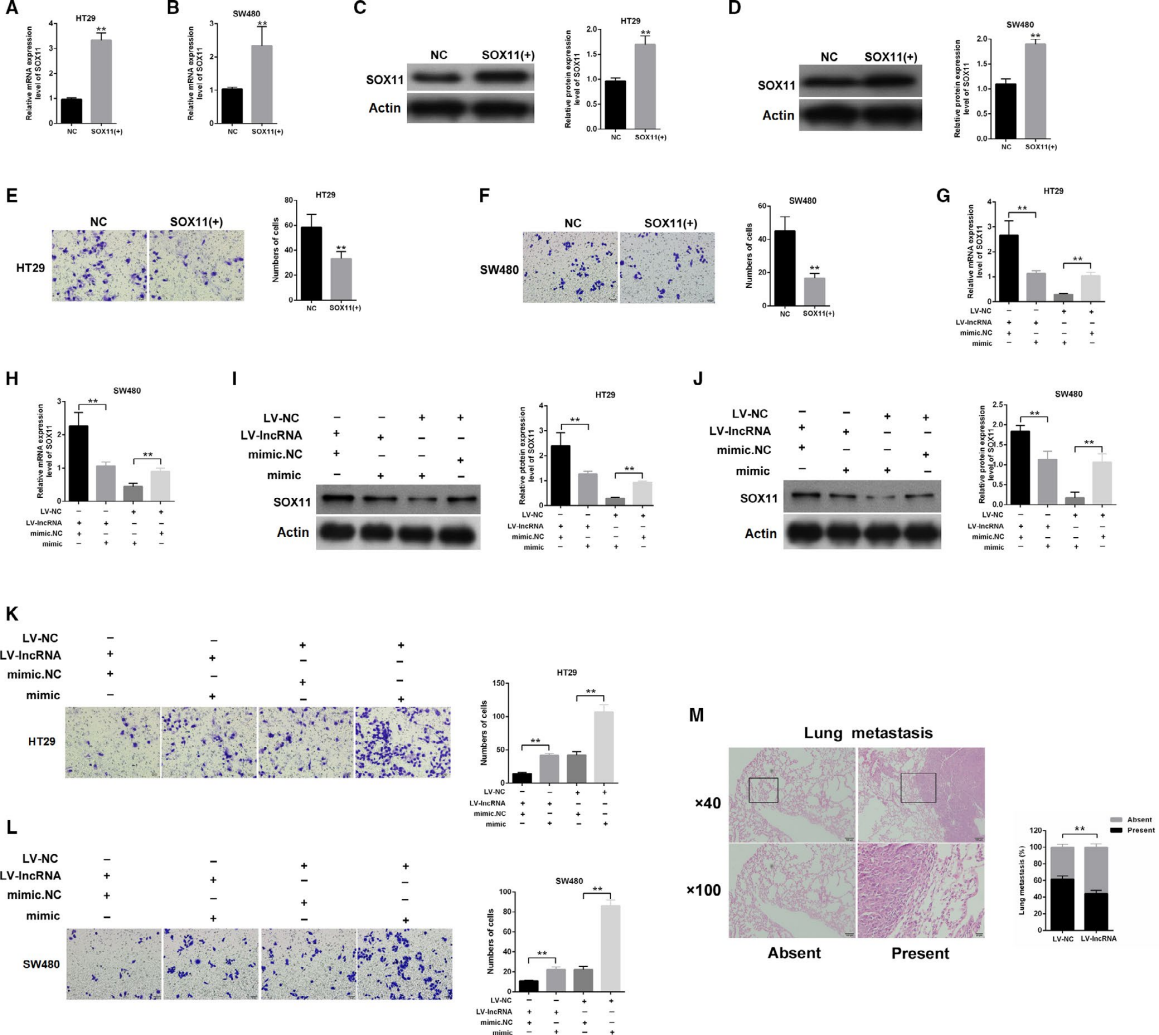
LINC00961 functions as anti‐oncogene by acting as miR‐223‐3p sponge. (A and B) The efficiency of pcDNA3.1‐SOX11 was determined by qPCR in HT29 and SW480 cells. (C and D) Western blot results showed that the expression of SOX11 was upregulated after transfection of pcDNA3.1‐SOX11. (E and F) Overexpression of SOX11 inhibited cell invasion in HT29 and SW480 cells. (G‐J) The mRNA and protein expression of SOX11 in HT29 and SW480 cells was determined by qPCR and western blot. Cells were transfected with LV‐ LINC00961 or mimic‐miR‐223‐3p. (K and L) The invasion capability of colon cells was determined by transwell assays. Cells were transfected with LV‐LINC00961 or mimic‐miR‐223‐3p. (M) Typical pictures of the lung metastasis of a mouse model. The percentage of the mice with or without metastatic nodules in the lungs was calculated. Each experiment was performed in triplicate. **P < .01 vs control

PcDNA3.1‐SOX11 was transfected into TH29 and SW480 cells to determine the effect of SOX11 on colon cancer cells. The efficiency of pcDNA3.1‐SOX11 in HT29 and SW480 was determined through qPCR and Western blot (Figure 5A–D). The results showed that SOX11 was successfully overexpressed in HT29 and SW480. Transwell results indicated that the overexpression of SOX11 significantly suppressed the invasion of cells in HT29 and SW480 (Figure 5E,5). A restore experiment was performed to explore the role of miR‐223‐3p in LINC00961 function. HT29 and SW480 was cotransfected with LV‐LINC00961 or mimic‐miR‐223‐3p. The qPCR results showed that mimic‐miR‐223‐3p could restore the mRNA expression level of SOX11 upregulation in colon cancer cell lines after transfection with LV‐LINC00961 (Figure 5G,H). Additionally, the western blot results indicated that mimic‐miR‐223‐3p could restore the protein expression level of SOX11 upregulation in HT29 and SW480 after transfection with LV‐LINC00961 (Figure 5I,J). Then, transwell assays were performed after cotransfection with LV‐LINC00961 or mimic‐miR‐223‐3p. The results of the restore experiment indicated that mimic‐miR‐223‐3p could restore the inhibition of the invasion of colon cancer cells after transfection with LV‐LINC00961 (Figure 5K,L). Lastly, HT29 cell line stably expressing LINC00961 was established. Lung metastasis models were established by injection of HT29 cells. The percentage of mice with or without metastatic nodules in the lungs was counted. As shown in Figure 5M, LINC00961 overexpression could suppress the metastasis ability of HT29. Thus, these results suggested that LINC00961 upregulated the SOX11 expression level and suppressed the invasion by acting as miR‐223‐3p sponge in colon cancer.

## 4 DISCUSSION

Cancer cell invasion and recurrence are the major causes of death among patients with colon cancer.^22^ Understanding the molecular mechanisms of the invasion and metastasis of colon cancer cells is the cornerstone of clinical treatments. However, the molecular mechanisms of colon cancer development are not fully understood. Numerous studies have investigated the potential roles of lncRNAs in colon cancer progression and have provided a new visual direction to reveal the potential mechanism in colon cancer.

In this research, LINC00961 was downregulated in colon cancer cells and tissues. LINC00961 was identified as a vital regulator in the invasion of several tumor cells. However, the potential roles in colon cancer remain unavailable. In present study, the roles of LINC00961 in colon cancer were investigated. The LINC00961 significantly suppressed the migration and invasion in HT29 and SW480. The mechanism of LINC00961 in colon cancer was further studied. Following the specific base pairing, lncRNAs contained the response elements of miRNA and function as competing endogenous RNAs (ceRNAs) to regulate mRNAs by competing for the shared response elements of miRNA. In‐depth studies have demonstrated that lncRNAs acted as a vital regulatory role in malignancies as competing endogenous RNAs. LncRNA TUG1 accelerates cells invasion in colorectal cancer via targeting miR‐600.^23^ LncRNA SNHG16 accelerates the cancer cells migration and invasion abilities through sponging miR‐520d‐3p and targeting STAT3 in hemangioma.^24^ HOTAIR facilitates the tumor progression in breast cancer cells via downregulation of miR‐20a‐5p and upregulation of HMGA2.^25^ A ceRNA network has been established in people with liver metastasis and those networks are helpful for the investigation of the vital regulators of colorectal cancer metastasis.^26^ Expression in cytoplasm is a necessary condition for the function as ceRNA. Our results showed that LINC00961 was mainly localized in the cytoplasm, suggesting that LINC00961 might function as ceRNA. MiR‐223‐3p was identified as the target miRNA of LINC00961. The relationship between LINC00961 and miR‐223‐3p was explored. QPCR results revealed that the miR‐223‐3p expression levels were decreased by LINC00961 upregulation but was increased by LINC00961 downregulation. Then, dual‐luciferase reporter assays were performed in HT29 and SW480. The luciferase activity in colon cells was decreased by mimic‐miR‐223‐3p but was increased by inhibitor‐miR‐223‐3p. However, the luciferase activity was not changed in the mutant‐type reporter gene after the transfection of mimic‐miR‐223‐3p or inhibitor‐miR‐223‐3p. Then, miR‐223‐3p possibly acts as a tumor promoter and enhances tumor cells metastasis via downregulation of SLC4A4 in renal cell carcinoma.^27^ MiR‐223‐3p inhibition relieves lung cancer development by targeting TGFBR3.^28^ MiR‐223‐3p inhibits tumor cell apoptosis and proliferation in testicular germ cell tumors.^29^ But the function of miR‐223‐3p in colon cancer is not fully studied. The results revealed that the inhibitior‐miR‐223‐3p weakened the migration and invasion in colon cancer cells. Targetscan was utilized to predict the potential target genes of miR‐223‐3p. SOX11 was predicted as a direct target gene of miR‐223‐3p by targetscan. Dual‐luciferase reporter assays showed that SOX11 was a direct target gene of miR‐223‐3p. SOX11 was identified as a cancer‐suppressor in the progression of different tumors. SOX11 promotes hepatocellular carcinoma cells apoptosis by the Wnt signaling pathway.^30^ SOX11 upregulation hinders the ovarian cancer cells invasion and proliferation abilities.^31^ However, the role of SOX11 in colon remains unavailable. SOX11 overexpression was successfully performed in colon cancer cells. Transwell assays indicated that SOX11 overexpression inhibited the cell invasion in HT29 and SW480. The relationship between LINC00961 and SOX11 was also investigated. The expression level of SOX11 was increased by LINC00961 overexpression but was decreased by LINC00961 silencing. The restore experiments were performed in colon cancer cells to investigate whether the miR‐223‐3p mediated the function of LINC00961. The restore experiment was conducted, and the cells were transfected with LV‐LINC00961 or mimic‐miR‐223‐3p. The results showed that miR‐223‐3p upregulation could restore the regulatory action of LINC00961. The pulmonary metastasis model in nude mice showed that LINC00961 overexpression inhibited colon cell metastasis.

## 5 CONCLUSION

LINC00961 inhibited the colon cancer cell invasion in vitro and in vivo. LINC00961 upregulated the expression level of SOX11 and inhibited the invasion by sponging miR‐223‐3p. Thus, LINC00961 might act as a potential diagnostic biomarker and therapeutic target for further clinical treatments.

All data are available upon request.

## CONFLICT OF INTEREST

None declared.

## AUTHORS’ CONTRIBUTIONS

HW, YD, JX, and CC designed the concept and experiments. HW, YD, DZ, XZ, and ZH performed the experiments, collected the data, and analyzed the results. HW wrote the manuscript. ZH, JX, and CC revised the manuscript.
